# C-X-C motif chemokine 12/C-X-C chemokine receptor type 7 signaling regulates breast cancer growth and metastasis by modulating the tumor microenvironment

**DOI:** 10.1186/bcr3665

**Published:** 2014-05-29

**Authors:** Nissar Ahmad Wani, Mohd W Nasser, Dinesh K Ahirwar, Helong Zhao, Zhenhua Miao, Konstantin Shilo, Ramesh K Ganju

**Affiliations:** 1 Department of Pathology, Comprehensive Cancer Center, The Ohio State University Wexner Medical Center, 460 West 12th Avenue, Columbus 43210, Ohio; 2 ChemoCentryx, Mountain View, California

## Abstract

**Introduction:**

Although C-X-C motif chemokine 12 (CXCL12) has been shown to bind to C-X-C chemokine receptor type 7 (CXCR7), the exact molecular mechanism regulations by CXCL12/CXCR7 axis in breast tumor growth and metastasis are not well understood. CXCR7 expression has been shown to be upregulated during pathological processes such as inflammation and cancer.

**Methods:**

Breast cancer cell lines were genetically silenced or pharmacologically inhibited for CXCR7 and/or its downstream target signal transducer and activator of transcription 3 (STAT3). 4T1 or 4T1 downregulated for CXCR7 and 4T1.2 breast cancer cell lines were injected in mammary gland of BALB/c mice to form tumors, and the molecular pathways regulating tumor growth and metastasis were assessed.

**Results:**

In this study, we observed that CXCL12 enhances CXCR7-mediated breast cancer migration. Furthermore, genetic silencing or pharmacologic inhibition of CXCR7 reduced breast tumor growth and metastasis. Further elucidation of mechanisms revealed that CXCR7 mediates tumor growth and metastasis by activating proinflammatory STAT3 signaling and angiogenic markers. Furthermore, enhanced breast tumorigenicity and invasiveness were associated with macrophage infiltration. CXCR7 recruits tumor-promoting macrophages (M2) to the tumor site through regulation of the macrophage colony-stimulating factor (M-CSF)/macrophage colony-stimulating factor receptor (MCSF-R) signaling pathway. In addition, CXCR7 regulated breast cancer metastasis by enhancing expression of metalloproteinases (MMP-9, MMP-2) and vascular cell-adhesion molecule-1 (VCAM-1). We also observed that CXCR7 is highly expressed in invasive ductal carcinoma (IDC) and metastatic breast tissue in human patient samples. In addition, high CXCR7 expression in tumors correlates with worse prognosis for both overall survival and lung metastasis-free survival in IDC patients.

**Conclusion:**

These observations reveal that CXCR7 enhances breast cancer growth and metastasis via a novel pathway by modulating the tumor microenvironment. These findings identify CXCR7-mediated STAT3 activation and modulation of the tumor microenvironment as novel regulation of breast cancer growth and metastasis. These studies indicate that new strategies using CXCR7 inhibitors could be developed for antimetastatic therapy.

## Introduction

Metastatic breast cancer is the most prevalent type of breast cancer worldwide and remains incurable despite recent therapeutic advances [1-3]. The significance of the CXCL12/CXCR4 axis in breast cancer invasion and metastasis has been widely investigated [4-8]. In addition to CXCR4, breast cancer cells express another chemokine receptor, CXCR7, which binds to CXCL12 with greater affinity than does CXCR4 [9]. Similar to chemokine signaling of CXCL12/CXCR4, CXCL12/CXCR7 signaling inhibits apoptosis and increases proliferation and metastasis in prostate cancer [10,11]. Mice genetically deficient in CXCR7 have abnormalities in cardiovascular and central nervous systems [12]. CXCR7 expression in non-small cell lung (NSCL) and breast cancer promotes their growth [13]. Breast cancer cells expressing CXCR7 mediate signaling through β-arrestin in a ligand-dependent manner rather than through G_i_or Ca_2+_ mobilization [14-16]. Cancer cells co-expressing CXCR4 and CXCR7 heterodimerize and mediate signaling, preferably through β-arrestin [14-16]. The exposure of CXCR4- and CXCR7-positive lymphoma cells to CXCL12 greatly potentiates their trans-endothelial migration, and this CXCL12-potentiated transendothelial migration is inhibited by blocking CXCR7 [17]. CXCR7 also plays an important role in vasculogenesis and angiogenesis through secretion of angiogenic factors [18,19]. One conflicting report regards CXCR7-mediated effects on breast tumor growth and metastasis, in which CXCR7 overexpression was shown to inhibit invasion and metastasis but enhanced primary tumor growth [18].

The STAT family of proteins are transcription factors, known for their role as integrators of cytokine and growth factor-receptor signaling, which is required for cell growth, survival, differentiation, and motility [20-22]. Activated STAT3 has also been shown to be associated with increased expression of cytokines, growth factors, matrix metalloproteinases (MMPs), and angiogenic factors [23]. In addition, STAT3 signaling modulates tumor growth and metastasis via recruitment of tumor-associated macrophages (TAMs) to the tumor site [24,25]. TAMs, which often constitute a major part of leukocyte infiltrates present in the tumor microenvironment, have been shown to enhance the tumor growth and metastasis of various cancers [26-28]. In addition, collaborative interactions of tumors with TAMs have been associated with poor prognosis in breast cancer [27,28]. Studies with mouse models have demonstrated that ablation of macrophages leads to inhibition of tumor progression and metastasis [29-31]. Cytokines/chemokines secreted by tumor cells activate TAMs, which in turn release factors that stimulate tumor cell proliferation, angiogenesis, incessant matrix turnover, and repression of adaptive immunity, which ultimately has a major impact on disease progression [30,32].

Although CXCL12 has been shown to bind to CXCR7, not much is currently known about the role of CXCL12/CXCR7 signaling in tumor growth and the early steps of metastasis within the primary tumors expressing CXCR7. In the present study, we sought to determine whether CXCR7 function controls tumor development *in vivo* and to determine the mechanism by which CXCR7 enhances breast cancer growth and metastasis. By using preclinical mouse models, we showed that a novel small-molecular-weight CXCR7-specific antagonist (CCX771) and STAT3-specific inhibitor (S31-201) inhibit breast cancer growth and metastasis. Further elucidation of molecular mechanisms revealed that CXCR7 enhances growth and metastasis via a novel pathway by modulating the tumor microenvironment. Moreover, high expression of CXCR7 in tumors correlates with worse prognosis for both overall survival and lung metastasis-free survival in IDC patients.

## Materials and methods

### Reagents

Cell-culture reagents were purchased from Gibco Laboratories (Grand Island, NY, USA). Chemokines were purchased from PeproTech. Anti-CXCR7 antibody was purchased from Abcam; VCAM-1, GAPDH, and pERK/ERK, from Santa Cruz; pSTAT3, from BD Biosciences; F4/80, CD11b, CD206, cyclin D1 and Ki67 from NeoMarkers. All other reagents were of standard grade. The small-molecule CXCR7 antagonists were obtained from ChemoCentryx, Inc.; and STAT3 inhibitor (S31-201) was purchased from Calbiochem, Billerica, MA.

### Cell culture

Mouse 4T1 breast cancer cell line and murine macrophage-like cell line (RAW 264.7) were purchased from American Type Culture Collection. The 4T1.2 breast cancer cells were obtained from Dr. Kang (Princeton University) after receiving permission from Dr. Anderson (Peter MacCallum Cancer Institute) [33]. The 4T1.2 clone was derived by single-cell cloning of 4T1 [34]. The 4T1.2 has been shown to be highly metastatic to lungs compared with 4T1 [34]. 4T1 Vector (4T1 Vec) and 4T1 downregulated for CXCR7 (4T1 sh-CXCR7) were obtained from ChemoCentryx, Inc. The 4T1 sh-CXCR7 cells showed 80% to -90% reduction in CXCR7 expression compared with vector control (Additional file 1: Figure S1). The cell lines were cultured in DMEM medium with 10% FBS, 5 units/ml penicillin, and 5 mg/ml streptomycin.

### Stimulation of cells

Cell stimulation was carried out as described earlier [35-37]. In brief, cells were serum starved for 4 hours at 37°C. Serum-starved cells were stimulated with 100 ng/ml CXCL12 and incubated at 37°C for various time periods. At the end of the stimulation, cells were harvested.

### Chemotaxis

The chemotactic assays were performed by using transwell chambers (Costar 8-μm pore size) [38]. Before the migration assay, cells were serum starved and pretreated with CCX771 (CXCR7 inhibitor) or S31-201 (STAT3 inhibitor III) or the appropriate vehicle control (DMSO) for 1 or 4 hours. A volume of 150 μl (1 × 10^6^ cells) from each sample was loaded onto the upper well. The medium (0.6 ml) with or without CXCL12 (100 ng/ml) was added to the lower well. The plates were incubated for 8 to 12 hours at 37°C in 5% CO_2_. After incubation, the porous inserts were removed, and the cells in the bottom chamber were stained and counted by using standard procedures. The results were expressed as the percentage of migrated cells as compared with the control (untreated cells) [38].

### Wound-healing assay

Wound-healing assays were performed as described previously [39,38]. Cells were grown to 70% confluence in complete DMEM. Monolayers were wounded by scratching with a sterile plastic 200-μl micropipette tip, washed, and incubated in DMEM (serum free) with CXCL12 (50 to 100 ng/ml) in the presence or absence of CXCR7 or STAT3 inhibitors. After 24 or 36 hours, cells were fixed with 4% paraformaldehyde in PBS for 5 minutes at RT and photographed by using a low-magnification phase-contrast microscope. The extent of migration into the wound area was evaluated qualitatively by using ImageJ software.

### Western blot analysis

Western blot (WB) analysis of lysates was done as described earlier [38-40]. Tumor samples or cells plated in 100 cm^2^ dishes were lysed in RIPA buffer. Then 50 μg of protein was loaded on 4% to –12% SDS–polyacrylamide gels (Invitrogen) under reducing conditions, transferred to nitrocellulose membranes (BioRad), and blocked with 5% milk in Tris-buffered saline and Tween 20 (TBST). Membranes were incubated overnight with primary antibody (1:1,000), washed 3 times with TBST, and incubated for 1 hour at RT with horseradish peroxidase-conjugated secondary antibody (1:4,000). Then the membranes were washed and stained by using a chemiluminescence system (ECL Amersham Biosciences) and exposed to X-ray film (Genemate).

### Orthotopic injection assay

The Ohio State University Administrative Panel on Laboratory Animal Care approved this study. Female BALB/c mice (6 to 8 weeks old) were anesthetized and injected with either 2.5 × 10^5^ murine 4T1 Vec or 4T1 sh-CXCR7 in 100 μl PBS or 1 × 10^5^ of 4T1.2 cells, 100 μl PBS, into the mammary gland (fourth mammary fat pad). After day 10, mice injected with 4T1.2 cells were injected subcutaneously with CXCR7-specific small-molecular-weight inhibitor CCX771 or STAT3 inhibitor (S31-201) at 5 mg/kg body weight, 3 times per week. Tumor growth was monitored weekly by using electronic calipers: tumor volume = (length × width^2^)/2. Mice were killed at the end of experiment, and tumors were excised and processed [4,38,40]. All mice were kept in the animal facility of Ohio State University in compliance with the guidelines and protocols approved by Institutional Animal Care and Use Committee (IACUC).

### FACS analysis

A single-cell suspension of the tumor-infiltrating cells was obtained as described [4,38,40]. For FACS analysis, freshly prepared tumor-infiltrating cells were incubated with anti-F4/80 PE, anti-CD11b APC, and anti-CD206 Alexa Fluor 488. After staining, the cells were analyzed with FACS Caliber by using CellQuest software (BD Biosciences).

### Immunohistochemistry

Tumors and lung samples were dissected from mice, fixed in formalin, and embedded in paraffin. Standard IHC techniques were used according to the manufacturer’s recommendations (Vector Laboratories) by using the primary antibodies against p-STAT3 (Abcam, 1:200), Ki67 (Neomarkers, 1:100), CD31 (Santa Cruz, 1:100), F4/80 (AbD Serotec, 1:50), and arginase1 (Santa Cruz (1:200) for overnight at 4°C. Vectastain Elite ABC reagents (Vector Laboratories) with avidin DH:biotinylated horseradish peroxidase H complex with 3,3′-diaminobenzidine (Polysciences) and Mayer hematoxylin (Fisher Scientific) were used for detection of the bound antibodies. The stained cells were counted in four different fields by using a bright-field microscope in each experimental group, and the average was calculated.

### Gelatin zymography

Gelatin zymography was performed with slight modifications, as described [4,38,40,41]. Cells were maintained at 80% confluency in serum-supplemented media. The monolayer was rinsed twice with PBS, and the cells were then kept under serum-free conditions. After 48-hour incubation at 37°C in 5% CO_2_, cell supernatants were collected and concentrated by using Centricon units (Millipore). Samples were resolved on 10% SDS–PAGE gels containing 0.3% gelatin. After electrophoresis, gels were washed and incubated with denaturing and developing buffers (Invitrogen). Subsequently, the gels were fixed and stained with Coomassie Brilliant Blue.

### Cancer patient survival analysis

Cancer patient survival prognosis was analyzed by using the Kaplan-Meier survival analysis coupled with a Logrank significance test. The IDC patient overall survival (OS) curve was generated based on The Cancer Genome Atlas (TCGA) database. Higher CXCR7 expression was defined as overexpression of *ackr3* (CXCR7 gene symbol), being greater than 1.0 fold of the standard deviation above the mean. The IDC patient lung metastasis-free survival curve was generated based on a breast cancer lung-metastasis study [42]. Lung metastasis-free survival (LMFS) was analyzed between 20 high-CXCR7 and 20 low-CXCR7 level patients.

### Statistical analysis

Statistical analysis was performed with Graphpad Prism software (Avenida de la Playa, La Jolla, CA, USA). The data were computed as mean ± SD. Group means were compared by using the Student *t* test. The acceptable level of significance was 5% for each analysis. For all graphs, *P < 0.05; **P < 0.01, ***P < 0.001. or ^#^*P* < 0.05; ^##^*P* < 0.01, and ^###^*P* < 0.001.

## Results

### CXCL12 induces CXCR7-dependent migration of breast cancer cells

**Figure 1:**
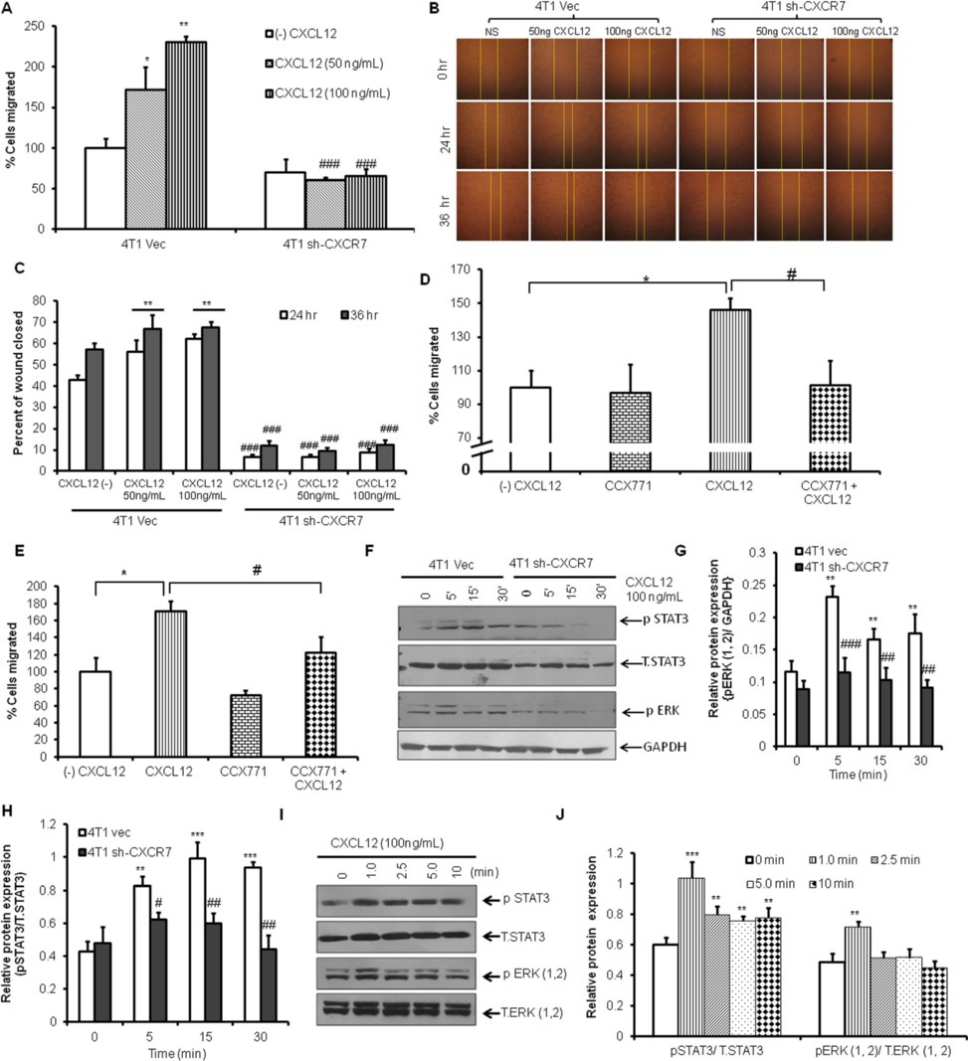
**CXCL12 enhances CXCR7-mediated cell migration and signaling. (A)** 4T1 Vec and CXCR7 shRNA-transfected cells were subjected to chemotaxis toward CXCL12 (50 and 100 ng/ml) by using the modified Boyden chamber assay, as described in Materials and methods. **(B)** 4T1 Vec and CXCR7 shRNA-transfected cells were grown to confluence in complete medium in six-well plates, and then a wound was made with a 200-μl pipette tip, and the closure of the wounds was monitored in the presence or absence of CXCL12 (50 and 100 ng/ml) by microscopy after 24 and 36 hours. **(C)** Quantitative analysis of percentage of wound closure. **(D)** 4T1 and **(E)** 4T1.2 cells were pretreated for 1 hour with vehicle or CCX771 (1 μ*M*) and were subjected to chemotactic assay in the absence or presence of CXCL12 (100 ng/ml). **(F)** 4T1 Vec and 4T1 sh-CXCR7 cells were serum starved for 4 hours and stimulated with CXCL12 (100 ng/ml) for different times, as indicated, and incubated at 37°C. After treatment, cells were washed, lysed, and analyzed with Western blotting for Phospho STAT3, STAT3, Phospho-ERK (p-ERK), and GAPDH by Immunoblotting. **(G, H)** Densitometry analysis of Western blots shows quantitation of pSTAT3 and pERK levels. **(I)** 4T1.2 cells were serum starved for 12 hours and stimulated with CXCL12 (100 ng/ml) for different time points, as indicated, and incubated at 37°C. After treatment, cells were washed, lysed, and analyzed for Phospho STAT3, STAT3, Phospho-ERK (p-ERK), and ERK with immunoblotting. **(J)** Densitometry analysis of Western blots shows quantitation of pSTAT3 and ERK levels, *P < 0.05, **P < 0.01, **P < 0.001 versus none, and ^##^*P* < 0.05, ^##^*P* < 0.01 ^###^*P* < 0.001 versus control.

The increased migratory ability of tumor cells determines their metastatic phenotype. The 4T1 and its highly metastatic clone 4T1.2 cell lines expressing CXCR7 were first evaluated for the role of CXCR7 in CXCL12-induced chemotaxis. As shown in Figure 1A through C, we found that downregulation of CXCR7 (Additional file 1: Figure S1) using sh-RNA significantly (p < 0.05) reduced CXCL12-induced migration and wound healing of 4T1 cells. However, the downregulation of CXCR7 did not seem significantly to inhibit the rate of proliferation of 4T1 cells (Additional file 2: Figure S2). Moreover, CXCL12-dependent increase in migration or wound-healing capability of 4T1 and 4T1.2 cells was significantly reduced in the presence of CXCR7 inhibitor (CCX771) (Figure 1D,E, and Additional file 3: Figure S3A,B). Taken together, these results suggest that CXCR7 enhanced CXCL12-induced migration of breast cancer.

### CXCL12/CXCR7 axis enhances breast cancer migration through activation of p44/p42 and STAT3 signaling pathways

Next, we analyzed CXCL12/CXCR7-mediated signaling mechanisms and observed a significant increase in phosphorylation of ERK (p44/p42) and STAT3 (S727) in CXCL12-stimulated 4T1 Vec cells compared with 4T1 cells downregulated for CXCR7 (Figure 1F-H). Moreover, we observed significant increases in ERK and STAT3 phosphorylation in 4T1.2 breast cancer cells on CXCL12 induction (Figure 1I,J). We did not observe any activation of p44/p42 ERK and STAT3 in 4T1 cells stimulated with TC-14012, which is a CXCR4-specific agonist (Additional file 4: Figure S4). STAT3 has been shown to be associated with proinflammatory responses and is activated in breast cancer tissues [22,43-47]. We further analyzed the role of CXCR7/STAT3 signaling in CXCL12-induced cell migration. We found that pharmacologic inhibition of STAT3 by S31-201 (S31-201 inhibits the STAT3 transcription factor by blocking the phosphorylation and dimerization events necessary for its activation) significantly reduced the CXCL12-induced migration of 4T1 cells as compared with control (Additional file 5: Figure S5), highlighting the role of CXCR7/STAT3 signaling in CXCL12-dependent migration of breast cancer cells. Taken together, these data suggest that p44/p42 ERK and STAT3 are downstream targets of CXCL12/CXCR7 signaling pathway.

### CXCR7 regulates breast cancer growth *in vivo*

**Figure 2:**
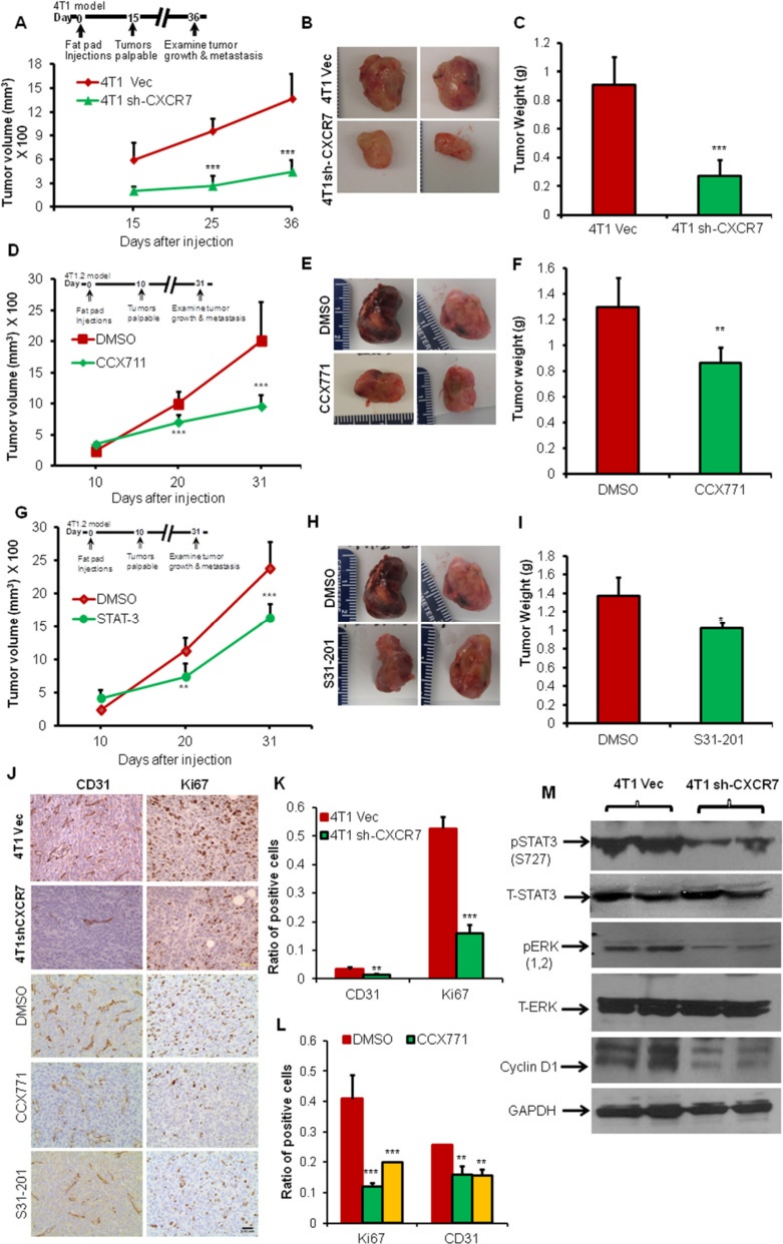
**CXCR7 regulates breast cancer tumor growth *in vivo *by regulating angiogenic, proliferative, and signaling pathways.** Female BALB/c mice (6 to 8 weeks old, *n* = 5) were anesthetized and injected with 2.5 x 10^5^ breast cancer cells 4T1 Vec and 4T1 sh-CXCR7 (downregulated for CXCR7). **(A)** Tumors were measured by digital calipers weekly for 36 days (mice model inset). **(B)** Representative photographs of tumors, 36 days after the injection of cells. **(C)** Tumor weight after 36 days. Female BALB/c mice (6 to 8 weeks old) were anesthetized and injected with 1 × 10^5^ viable 4T1.2 cells into the fourth mammary fat pad. Mice were divided into three groups of five mice each and were injected intraperitoneally with either DMSO or CXCR7 inhibitor (CCX771) or STAT3 inhibitor (S31-201) at 5 mg/kg body weight 3 times a week for 21 days (inhibitors were injected in mice after tumors were palpable), **(D, G)** tumors were measured with digital calipers (mice model inset) **(E, H)** Representative photographs of tumors, 31 days after the injection of cells. **(F, I)** Tumor weight after 31 days. **(J)** 4T1 Vec, 4T1 sh-CXCR7, and 4T1.2 cell line-derived tumors from CCX771 or S31-201 treated or untreated group were subjected to immunohistochemical (40×) staining with anti-CD31 or Ki67. **(K, L)** The stained cells were counted in four different fields by using bright-field microscope in each experimental group and the average was calculated. Bars represent the mean ± SD of number of CD31-positive blood vessels and Ki67-positive cells to that of total cells. **(M)** 4T1 Vec and 4T1 sh-CXCR7 cell line-derived tumors were lysed and analyzed for Phospho STAT3, STAT3, Phospho-ERK (p-ERK), ERK, cyclin D1, and GAPDH with immunoblotting. Data represent the mean ± SD per experimental group. Scale bars, 0.03 mm. *P < 0.05, **P < 0.01, ***P < 0.001 versus control.

To evaluate the effects of CXCR7 on the growth of breast cancer cells *in vivo*, we implanted 4T1 Vec control or 4T1 sh-CXCR7 or 4T1.2 cells into the mammary fat pad of BALB/c mice. We injected mice bearing 4T1.2 tumors with CCX771 or S31-201 at 5 mg/kg body weight, 3 times a week. Similar to the results of Miao *et al.*[13], both tumor volume as well as tumor weight were reduced in mouse xenografts of 4T1 sh-CXCR7 cells compared with vector control cells (Figure 2A-C). Moreover, a significant decrease occurred in the 4T1.2-derived tumor volume and weight of mouse injected with CCX771 (Figure 2D*-*F) or with S31-201, compared with respective controls (Figure 2G-I). Collectively, these observations demonstrate that CXCR7 promotes breast cancer tumor growth *in vivo*.

There was decreased Ki67 expression (Figure 2J-L) and hence reduced mitotic index in the CXCR7 downregulated or inhibitor-treated tumors compared with respective controls (Figure 2J-L). Microvasculature was more developed in tumors formed by 4T1 Vec control or 4T1.2 cells compared with CXCR7 downregulated or inhibitor-treated tumors, as shown by increased CD31 expression and larger size of capillaries (Figure 2J-L). We observed that STAT3 and ERK signaling pathways are downstream targets of CXCL12/CXCR7 pathway. Therefore, we analyzed the expression of phospho-ERK as well as phospho-STAT3 and its downstream target cyclin D1 in mouse tumors. We observed that the tumors of mice downregulated for CXCR7 showed reduced activation for p44/p42 ERK and STAT3 and cyclin D1 (Figure 2M). Moreover 4T1.2-derived tumors of mice injected with CXCR7 or S31-201 showed reduced p-STAT3 activation compared with respective control (Additional file 6: Figure S6). These results indicate that CXCR7 promotes tumor growth through activation of certain proinflammatory, cell-cycle progression, and angiogenic signaling pathways.

### CXCR7 promotes breast cancer metastasis

**Figure 3:**
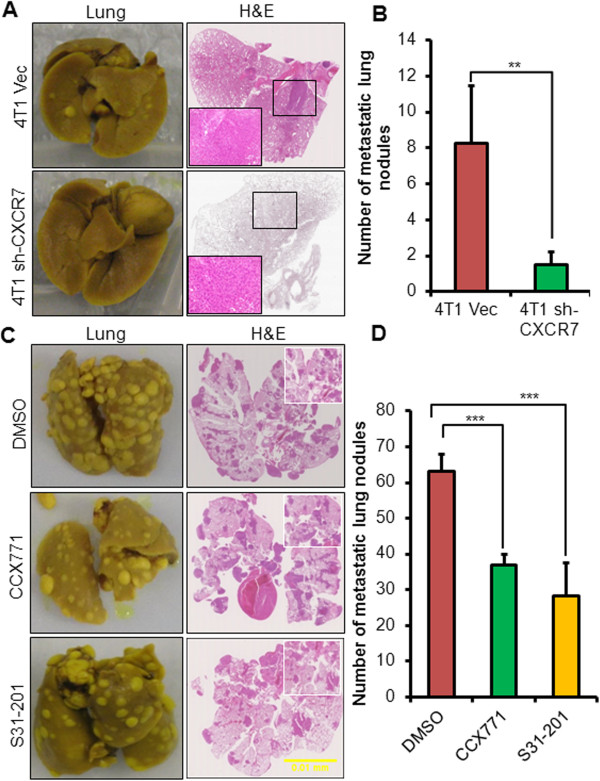
**CXCR7 promotes breast cancer metastasis.** Lungs were removed from mice used in the experiment presented in Figure 2 and inflated with Bouin fixative and number of metastatic nodules on the lungs was counted with the aid of a dissecting microscope. **(A)** H&E (2.5×) staining of metastatic nodules in the lungs of mice bearing 4T1 Vec or 4T1 sh-CXCR7 (insets: 20× magnification of the areas selected by rectangles) **(B)** bar showing the number of metastatic lung nodules and **(C)** H&E (2.5X) staining of metastatic nodules in the lungs of mice bearing 4T1.2 tumors treated with either DMSO or CXCR7 inhibitor (CCX771) or STAT3 inhibitor (S31-201) insets: 5× magnification and **(D)** bar showing the number of metastatic lung nodules. Scale bars, 0.01 mm. **p < 0.01, ***p < 0.001 versus control.

We also analyzed the role of CXCR7 in breast cancer metastasis. Genetic silencing of CXCR7 in 4T1 cells significantly reduced the number of lung metastatic nodules in mice (Figure 3A,B). Similarly, pharmacologic inhibition of CXCR7 or its downstream target STAT3 showed significant reduction in lung metastasis in mice bearing 4T1.2 tumors (Figure 3C,D). These studies demonstrate that CXCR7 regulates breast cancer metastasis to the lungs.

### CXCR7 modulates tumor microenvironment

**Figure 4:**
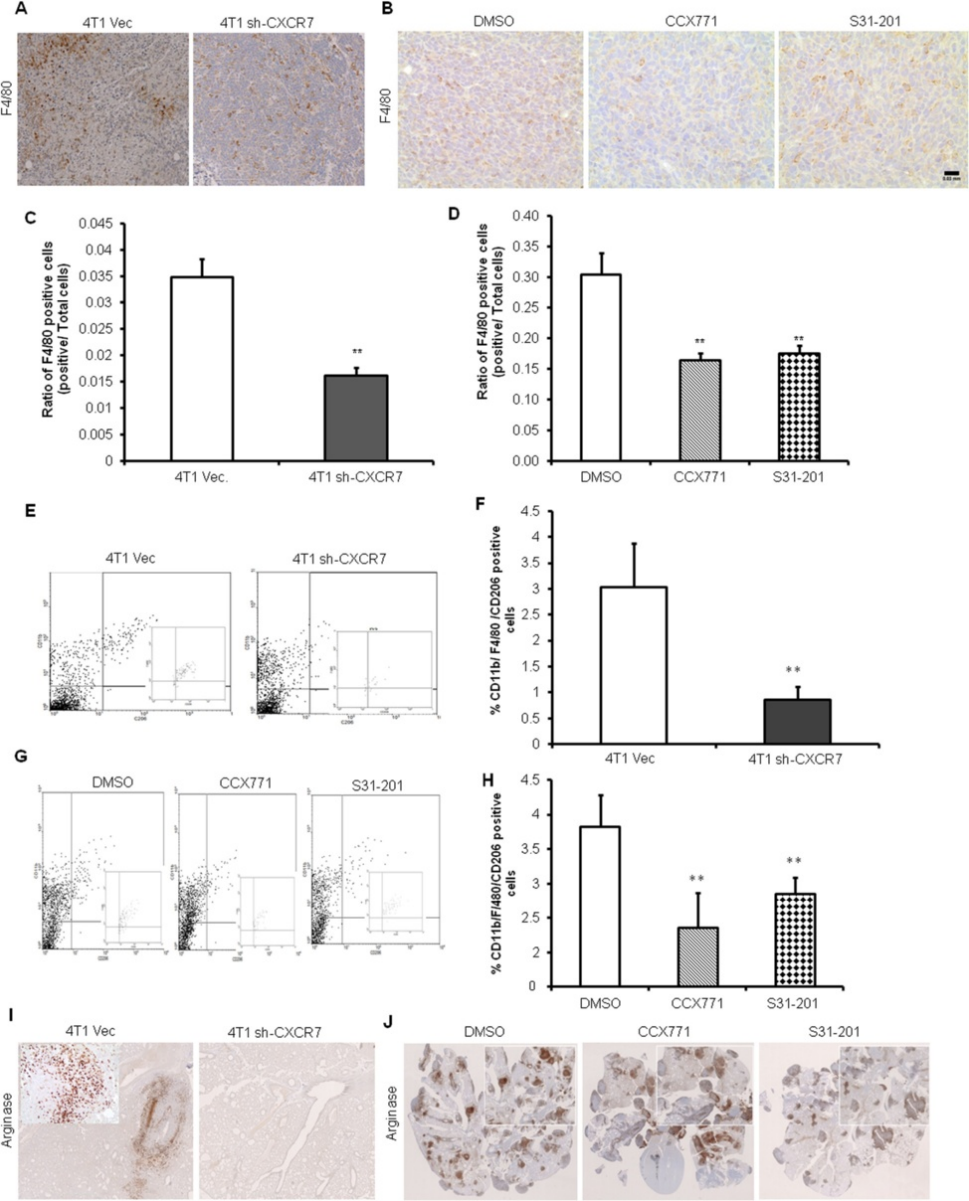
**CXCR7 enhances recruitment of M2 macrophages into tumor stroma.** Tumors from mice used in experiment presented in Figure 2 were subjected to IHC (20×) staining for macrophage marker, F4/80. **(A)** Representative image of 4T1 Vec and 4T1 sh-CXCR7 **(B)** 4T1.2 tumors treated with either DMSO or CXCR7 inhibitor (CCX771) or STAT3 inhibitor (S31-201). The F4/80^+^-stained cells were counted in four different fields by using bright-field microscope in each experimental group, and the average was calculated. Bars represent the mean ± SD of number of F4/80 macrophages **(C)** 4T1 Vec, 4T1 sh-CXCR7 and **(D)** 4T1.2 tumors treated with either DMSO or CXCR7 inhibitor (CCX771) or STAT3 inhibitor (S31-201). CD11b^+^F4/80^+^CD206^+^ cells in tumors were quantified by flow cytometry **(E, F)** 4T1 and 4T1 sh-CXCR7 **(G, H)** 4T1.2 tumors treated with either DMSO or CXCR7 inhibitor (CCX771) or STAT3 inhibitor (S31-201). Lungs were removed and stained for Arginase-1 (5X) **(I)** 4T1 Vec or 4T1 sh-CXCR7 (insets: 40× magnification) and **(J)** 4T1.2 tumors treated with either DMSO or CXCR7 inhibitor (CCX771) or STAT3 inhibitor (S31-201) (2.5×; insets, 10× magnification). Data represent the mean ± SD per experimental group. Scale bars, 0.03 mm. *P < 0.05, ^**^P < 0.01 versus vehicle or vector control.

The tumor microenvironment is characterized by a reactive stroma with an abundance of inflammatory mediators and leukocytes, dysregulated vessels, and proteolytic enzymes. TAMs have been shown to be a major component of tumors infiltrates [28,30]. Macrophages exhibit protumoral functions through the promotion of angiogenesis and enhancement of tumor-cell migration and invasion [28]. Therefore, 4T1- and 4T1.2-derived primary tumors were evaluated with IHC for macrophage marker F4/80. F4/80^+^ macrophages were significantly reduced in CXCR7 downregulated or inhibitor (CXCR7 or STAT3)-treated tumors compared with vector control or vehicle control, respectively (Figure 4A-D). TAMs can be divided into two main classes: tumor-suppressive M1 (classically activated) and tumor-promoting M2 (alternative). Macrophages M1 are characterized by expression of iNOS, whereas M2 macrophages have a decreased level of iNOS and are identified by their signature expression of arginase-1 (Arg-1) and mannose receptor (CD206) [40,48]. We used flow cytometry to quantify immune cell infiltrates in digested tumors harvested from mammary fat pads. The CD11b^+^/F4/80^+^/CD206^+^ macrophages were significantly reduced in CXCR7 downregulated or inhibitor-treated tumors compared with vector control or vehicle control, respectively (Figure 4E-H). We further confirmed increased M2 phenotype in the lungs of mice with metastatic tumors by analyzing Arg-1 expression. We observed higher Arg-1-positive macrophages associated with tumors in lungs compared with mice bearing CXCR7-downregulated or CXCR7 inhibitor-treated tumors (Figure 4I).

The lungs were also stained for p-STAT3 expression, which showed reduced STAT3 activation in mice bearing CXCR7-downregulated or inhibitor-treated tumors compared with vector or vehicle control (data not shown).

### CXCR7 modulates tumor microenvironment by enhancing expressions of MCS-F, MMPs, and VCAM-1

**Figure 5:**
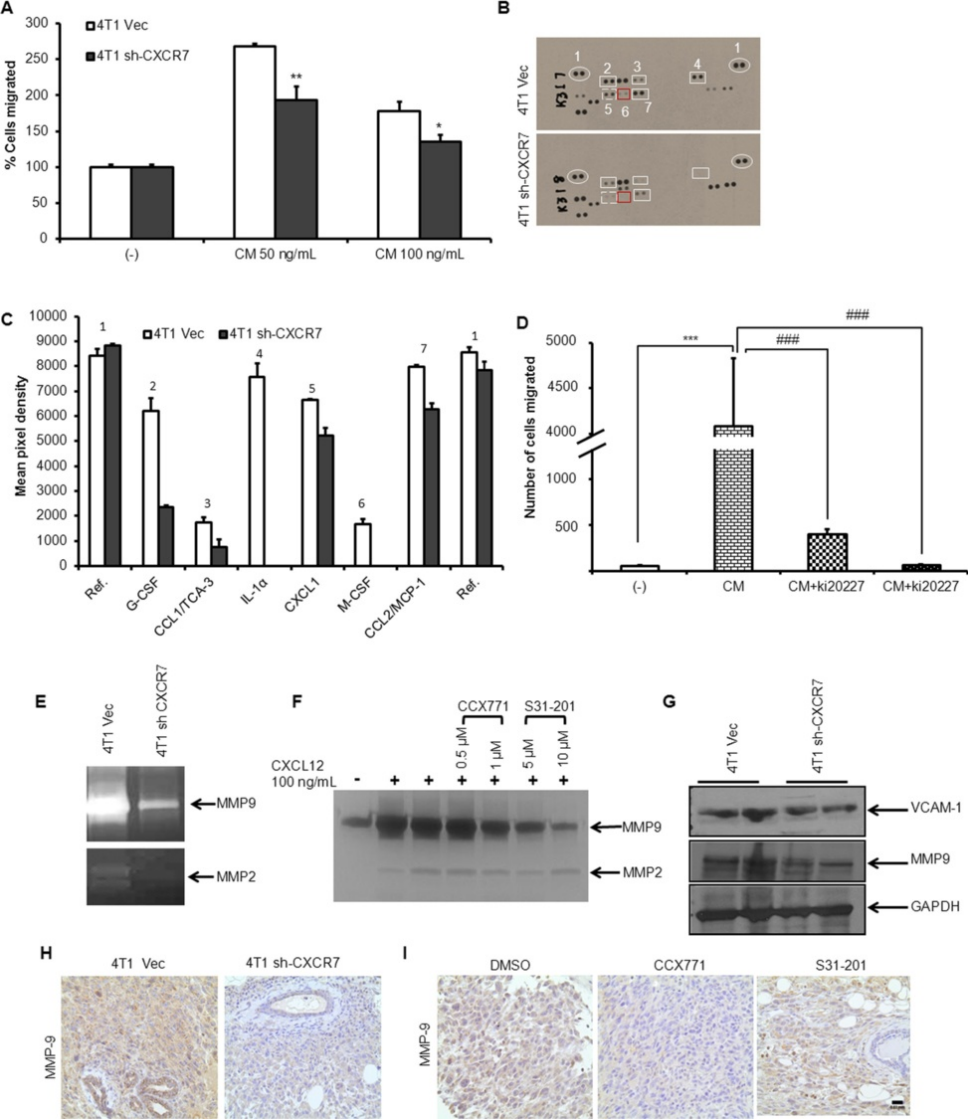
**CXCR7 regulates M-CSF, MMPs, and VCAM-1 expression. (A)** Murine macrophages (RAW 264.7) were serum starved for 4 hours and plated on the top chamber of 8-μm-pore polycarbonate membrane filters and SFM containing 4T1 or 4T1 sh-CXCR7 CM (50 to 100 μg/ml) was placed in the lower chamber. After 12 hours of incubation, cells that migrated across the filter toward SFM with or without CM (50 to 100 μg/ml) were fixed, stained, and counted by using bright-field microscopy in five random fields. **(B)** Conditioned media (CM) obtained from Vector or CXCR7-downregulated cells were subjected to cytokine profiling. **(C)** The array data were quantitated by ImageJ to generate a protein profile (histogram) **(D)** Murine macrophages (RAW 264.7) were serum starved for 4 hours and treated with 50 and 100 μ*M* ki20227 (MCSF-R inhibitor) and then plated on the top chamber of 8-μm-pore polycarbonate membrane filters. SFM, in the absence or presence of 4T1 CM (100 μg/ml), was placed in the lower chamber. After 12 hours of incubation, cells that migrated were fixed, stained, and counted by using bright-field microscopy in five random fields. **(E)** CM obtained from vector or CXCR7-downregulated cells was subjected to gelatin zymography for MMPs activity. **(F)** CM obtained from 4T1.2 cells treated with CXCR7-specific (CCX771) at 0.5 and 1 μ*M* concentration or STAT3-specific inhibitors (S31-201) at 5 and 10 μ*M* concentration in the presence and absence of CXCL12 (100 ng/ml) were subjected to gelatin zymography. **(G)** 4T1 and 4T1 shCXCR7 cell line-derived tumors were lysed and analyzed for VCAM-1, MMP-9, and GAPDH expression by Immunoblotting. The 4T1 Vec, 4T1 sh-CXCR7 **(H),** and 4T1.2 **(I)** cell line-derived tumors from CCX771 or S31-201 treated or untreated groups were subjected to immunohistochemical staining for MMP-9 expression (40×). Data represent the mean ± SD per experimental group. Scale bars, 0.03 mm. ^**^P < 0.01, ^***^P < 0.001 versus none and ^###^*P* < 0.01 versus control.

Next, we analyzed the CXCR7-mediated mechanisms that modulate tumor microenvironment. We analyzed the effect of factors secreted into the conditioned media (CM) by breast cancer cells on chemotaxis of murine macrophage-like cells (RAW 264.7). We observed a significant reduction in chemotaxis of macrophages on stimulation with the CM of 4T1 sh-CXCR7 cells, compared with that of vector control cells (Figure 5A). The cytokine array of CM from 4T1 Vec and 4T1 sh-CXCR7 cells revealed that 4T1 sh-CXCR7 cells secrete lower levels of macrophage colony-stimulating factor (M-CSF) compared with vector control cells (Figure 5B,C). The M-CSF has been shown to be the main factor responsible for the monocyte-macrophage recruitment to the tumor site [49,50]. The inhibition of macrophage colony-stimulating factor receptor (MCSF-R) in macrophages by ki20227 (an inhibitor of the M-CSF receptor (c-Fms)) reduced their migration toward the CM of 4T1 breast cancer cells (Figure 5D). These results suggest that CXCR7 might regulate the secretion of M-CSF, which may be important in recruiting M2 macrophages to the tumor site.

MMPs are known to degrade extracellular matrix (ECM) proteins in the cellular microenvironment and to promote tumor progression [51,52]. We observed reduced MMP-9 and MMP-2 activity in 4T1 sh-CXCR7 cells as compared with vector control (Figure 5E), and pharmacologic inhibition of 4T1.2 cells with CXCR7- or STAT3-specific inhibitors significantly reduced secretion of MMP-9 in these cells compared with vehicle (Figure 5F). Moreover, mice bearing 4T1 sh-CXCR7 tumors or tumors treated with CXCR7 or STAT3-inhibitor showed reduced expression of MMP-9 compared with respective controls (Figure 5G-I). Aberrant expression of vascular cell-adhesion molecule-1 (VCAM-1) primes metastatic cells for survival and outgrowth in the leukocyte-rich lung-parenchyma microenvironment [53]. We observed reduced expression of VCAM-1 in tumors of mice downregulated for CXCR7 (Figure 5F).

### CXCR7 is overexpressed in breast cancer patients and is associated with worse clinical outcome

**Figure 6:**
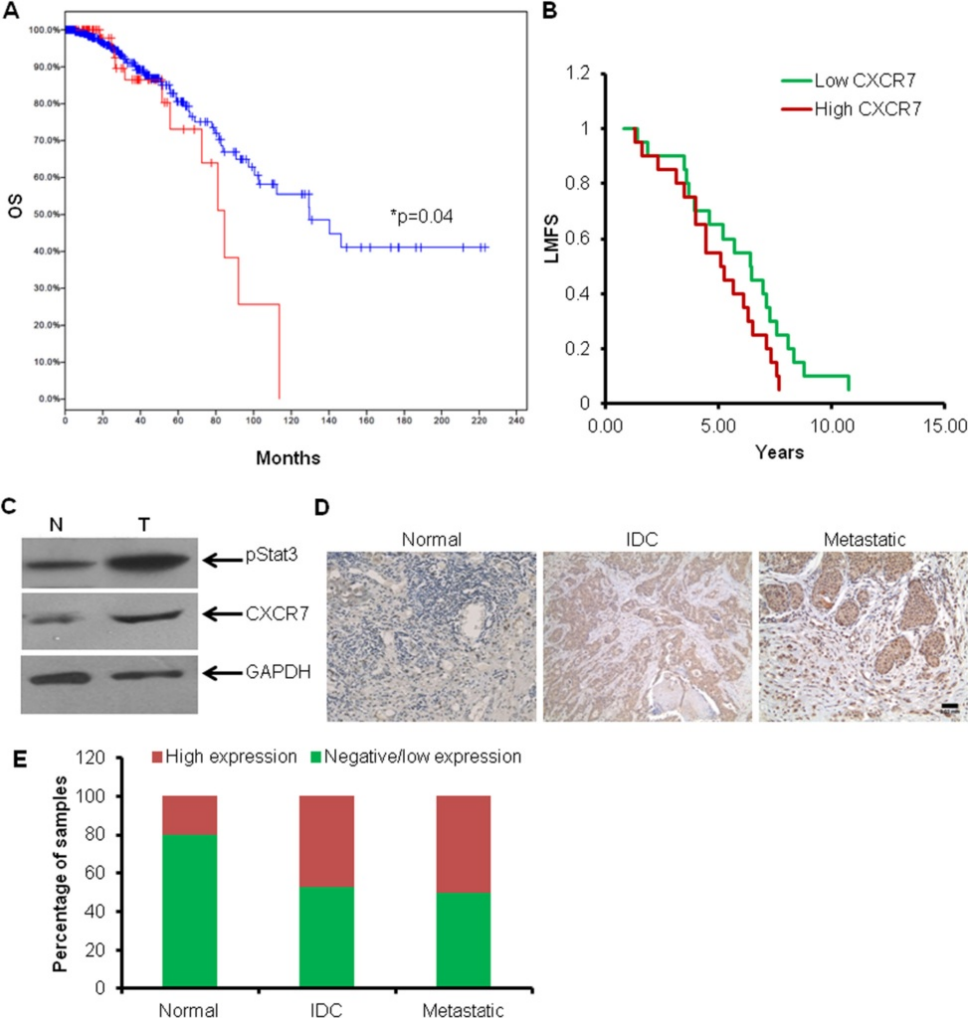
**CXCR7 expression in human breast tumors correlates with worse patient outcome and CXCR7 and STAT3 expression in breast cancer patients. (A)** Overall survival (OS) of TCGA IDC patients with or without *ackr3* overexpression (greater than 1.0-fold SD above mean). **(B)** Lung metastasis-free survival (LMFS) of IDC patients from study GSE2603. LMFS was compared between 20 high-CXCR7- and 20 low-CXCR7-expressing patients. **(C)** Breast tumors and adjacent normal tissue (*n* = 4) were lysed with RIPA buffer, and the lysates were analyzed with Western blotting by using specific antibodies against p-STAT3 (86 kDa), CXCR7 (52 kDa), and GAPDH (37 kDa) as loading control. **(D, E)** Representative tissue microarray cores of normal and cancerous breast tissue (20×, scale bar, 0.03 mm). Tissue microarray (TMA) samples containing 10 normal, 10 metastatic, and 38 invasive ductal carcinomas (IDCs) were analyzed with immunohistochemistry by using CXCR7 antibodies (kindly provided by ChemoCentryx).

To test whether CXCR7 expression in breast cancer patients correlates with clinical outcome, we first analyzed the publically available RNA array datasets. Analysis of TCGA (The Cancer Genome Atlas) IDC breast cancer database revealed that overexpression of CXCR7 correlates with worse overall survival (OS) [54,55]. In total, 16.2% of the 525 IDC patients with CXCR7 overexpression had a prognostic median survival of 84.53 months, compared with 129.61 months for the rest of the patients (Figure 6A). In another breast cancer lung-metastasis study (Gene Expression Omnibus accession GSE2603), patients with high CXCR7 expression had worse prognosis for lung metastasis-free survival (LMFS) compared with those with low CXCR7 (Figure 6B). We also examined CXCR7 expression in human breast tumor samples with Western blotting. As expected, CXCR7 was highly expressed in breast cancer patients (Figure 6C). We further analyzed CXCR7 expression in breast tissue microarray (TMA) with IHC and observed that its expression was higher in patients with lymph node metastasis as compared with normal samples (Figure 6D,E). Taken together, our data indicate that CXCR7 expression in the human breast tumors predicts worse outcomes, and its expression is higher in invasive and metastatic breast cancer patients.

## Discussion

Chemokines and their cognate receptors are extensively involved in cancer metastasis [56]. Metastatic breast cancer is the leading cause of cancer-related death in women worldwide, and understanding of the mechanism that facilitates metastatic tumor progression is of great importance. In this regard, the role of the CXCL12/CXCR4 axis in breast cancer invasion and metastasis is widely studied [40,57,58]. In addition to CXCR4, breast cancer cells express another chemokine receptor, CXCR7, which binds to CXCL12 and introduces a new level of complexity in chemokine-receptor signaling. Although CXCL12 signaling has been implicated in breast cancer metastasis as a homing mechanism for cancer cells to the metastatic sites, not much is currently known about the role of CXCL12 signaling in the early steps of metastasis within the primary tumor expressing CXCR7 and the mechanisms by which CXCR7-mediates breast cancer growth and metastasis.

Conflicting reports with respect to role of CXCR4 and CXCR7 have been made [9,59-61]. Some support the role of both CXCR4 and CXCR7 in breast cancer growth [15], whereas another study highlights the role of CXCR7 in inhibiting invasion and metastasis of breast cancer [9,59-61]. The differences in the results observed with regard to CXCR7 by different groups might be due to different cell types used or differences in experimental conditions and/or different model systems, which might be the result of differences in expression levels of CXCR4 and CXCR7. These receptors dimerize into homo- and hetero- forms *in vivo,* and the ability to form homo- or heterodimers seems to depend on the expression levels of both these receptors [12,62,63].

Keeping these things in view, the present study was designed with the aim to determine the role of CXCR7 in breast cancer growth and metastasis and to delineate the mechanistic insights into how CXCR7 regulates breast cancer growth and metastasis. CXCL12/CXCR7 signaling has been shown to inhibit apoptosis and increase proliferation [13,64,65]. Mice genetically deficient in CXCR7 have abnormalities in their cardiovascular and central nervous systems [12]. CXCR7 expression in NSCL and breast cancer is correlated with lymph node metastasis and poor prognosis [66,67].

First, we observed that 4T1 and 4T1.2 cells express CXCR7. For downregulation of CXCR7, we used sh-RNA against CXCR7, which significantly reduced CXCR7 expression in 4T1 cells. The 4T1.2 cells, subclones of 4T1, was difficult to transfect, but because it is highly metastatic, we used it for experiments that involved inhibitors. Our results revealed the role of the CXCL12/CXCR7 axis in regulating cell migration and wound healing in breast cancer cells, which have been shown to play an important role in regulating breast cancer metastasis. We elucidated CXCR7-mediated signaling pathways and showed that CXCL12 induced p44/p42 ERK and p-STAT3 in breast cancer cells. ERK has been shown to regulate migration in several cell types [45,47].

Moreover, the aberrant activation of STAT3 has been broadly characterized as a regulator of tumorigenesis through its effects in tumor cells, tumor microenvironment, and metastasis [68-71]. The elevated levels of STAT3 phosphorylation have been shown to be associated with regulation of apoptosis, cell-cycle progression, and tumor angiogenesis in invasive breast cancer tissues [25]. STAT3 has been shown to be constitutively activated in 35% to 60% of breast cancers [24,72]. We showed that downregulation or inhibition of CXCR7 or STAT3 reduced breast tumor growth and spontaneous metastasis in the orthotopic model by regulating proliferative and angiogeneic pathways.

Further elucidation of mechanisms revealed that CXCR7 may enhance growth and metastasis through recruitment of M2 (TAMs) macrophages. TAMs promote cancer metastasis through several mechanisms, including the promotion of angiogenesis [73], induction of tumor growth [74], and enhancement of tumor cell migration and invasion. We showed that M2-specific markers were reduced in tumors genetically silenced or pharmacologically inhibited for CXCR7 or STAT3. Furthermore, decreased migration, but not proliferation of RAW 264.7 cells toward the CM of CXCR7-downregulated cells confirmed our *in vivo* finding of reduced recruitment of macrophages in CXCR7-downregulated tumors. TAMs are recruited to tumors by growth factors and chemokines, which are often produced by the cancer and stromal cells in the tumor site. We showed that CXCR7 regulates the secretion of M-CSF, which has been shown to correlate with increased TAM numbers in various human tumors [28,75]. Blocking of M-CSF receptor in macrophages reduced their migration toward CM of 4T1 cells. Therefore, CXCR7 might play an important role in recruitment of macrophages through modulation of the M-CSF/MCSF-R pathway.

A critical step in cancer cell metastasis is the degradation of extracellular matrix components by MMPs, permitting malignant cells to separate from the primary tumor and access circulatory conduits for seeding at distant organs. We observed reduced MMP-9 and MMP-2 activity in CM and tumors of mice downregulated or inhibited for CXCR7, suggesting that CXCR7 positively correlates with MMP(s) secretion in breast cancer cells, or the reduced recruitment of TAMs to the tumor site might be responsible for less secretion of MMPs at the tumor site, thereby reducing the migration of breast cancer. Breast cancer cells expressing the leukocyte receptor VCAM-1 can thrive in leukocyte-rich microenvironments through juxtacrine activation of a VCAM-1–Ezrin-PI3K/Akt survival pathway [53]. The decreased expression of VCAM-1 in CXCR7-downregulated cells may lead to its reduced interaction with macrophages, as observed by lesser numbers of TAMs in the lungs of mice bearing tumors silenced or inhibited for CXCR7 or its downstream target, STAT3. Reduced VCAM-1 expression in tumors, along with reduced recruitment of macrophages to the tumor site, may therefore be responsible for decrease in tumor cell metastasis to the lungs of mice bearing CXCR7-downregulated tumors. These results suggest that CXCR7 may enhance tumor growth and metastasis by recruiting M2 macrophages to the tumor site and regulating the secretion and expression of MMPs and VCAM-1 in breast cancer cells. These properties might provide the tumor cells with a survival advantage, allowing them to reach and colonize in lungs or other metastasis-prone areas within the body.

We have shown that CXCR7 is highly expressed in invasive and metastatic tumors. Importantly, the overexpression of CXCR7 in human breast tumors correlates with worse clinical outcome. Thus, our data from mouse models and human samples suggest that CXCR7 may be used as a prognostic marker for metastatic breast cancer.

## Conclusion

**Figure 7:**
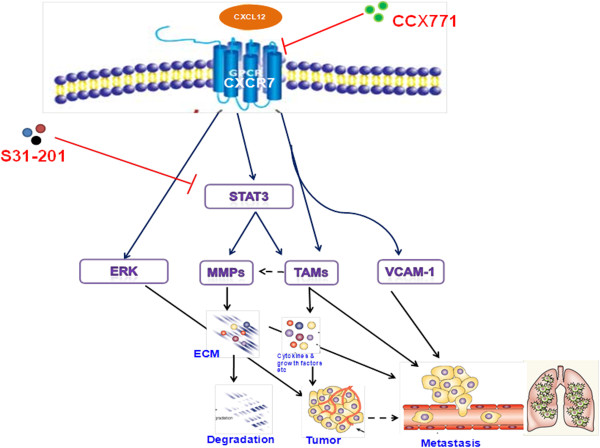
**Schematic representation of CXCR7-mediated signaling that regulates breast cancer growth and metastasis.** CXCL12 binding to CXCR7 leads to activation of ERK and STAT3 and enhanced expression of VCAM-1. CXCR7 also, either directly or indirectly through STAT3, may enhance MMP-9 and TAMs recruitment to the tumor site. TAMs in turn enhance growth factor, chemokines, and MMPs secretion in a tumor microenvironment. These CXCR7-mediated mechanisms may regulate primary breast tumor growth and metastasis, especially to lungs.

In summary (Figure 7), our studies revealed that CXCL12/CXCR7-induced STAT3-mediated pathways may enhance tumor growth by regulating angiogenic and proliferative pathways. They may also regulate metastasis by recruiting TAM(s), by enhancing the secretion of M-CSF, MMP-2 and 9, and enhancing VCAM-1 expression. These studies indicate that CXCR7 may enhance tumor growth and metastasis through modulation of the tumor microenvironment by enhancing recruitment of TAM, thus activating certain proinflammatory, angiogenic, and metastatic pathways.

Importantly, overexpression of CXCR7 predicted poor clinical outcome in a cohort of breast cancer patients, suggesting that blocking CXCR7 signaling may be a potential therapeutic approach to inhibit highly metastatic and invasive breast cancer, which is a major cause of mortality in breast cancer patients. In addition, these studies indicate that small-molecular-weight inhibitors against CXCR7 could be developed for antimetastatic therapies.

## Abbreviations

ERK: Extracellular signal-regulated kinases; IDC: invasive ductal carcinoma; MMPs: matrix metalloproteinases; STAT3: signal transducer and activator of transcription 3; TAM: tumor-associated macrophage; TMA: tissue microarray; VCAM-1: vascular cell-adhesion molecule-1.

## Competing interest

No potential conflict of interest in relation to this article exists.

## Authors’ contributions

NAW designed and performed the studies and drafted the manuscript. KS helped in IHC analysis of tissue samples. ZM generated 4T1 stable cell lines. HZ, MWN, and DKA assisted in performing experiments and statistical analysis. MWN also helped in drafting the manuscript. RKG conceived of the study and was involved in its design and coordination and helped in drafting the manuscript. All authors contributed to critical analysis and approval of the manuscript.

## Supplementary Material

Additional file 1: Figure S1 CXCR7 expression in 4T1 Vec and sh-RNA downregulated cells. 4T1 Vec and 4T1 sh-CXCR7 cell lines were lysed and analyzed with Western blotting for CXCR7 and GAPDH expression. Data represent the mean ± SD per experimental group. ***P < 0.001 versus vector control. Click here for file

Additional file 2: Figure S2 CXCR7 downregulation did not inhibit proliferation *in vitro*. 4T1 Vec and 4T1 sh-CXCR7 cell lines were seeded at a density of 5,000 cells per well in 96-well plates and allowed to grow for 24 to 48 hours in SFM. Cell viability was measured by using the MTT assay (Roche), based on the absorbance reading at 570 nm with respect to the control. Click here for file

Additional file 3: Figure S3 CXCL12 enhances CXCR7- mediated cell migration. 4T1 **(A)** and 4T1.2 **(B)** cells treated with CCX771 (1 μ*M*) were grown for confluence in incomplete medium in six-well plates, and then a scratch was made with a 200-μl pipette tip to make wounds; the closure of the wounds was monitored in the presence or absence of CXCL12 (100 ng/ml) by microscopy after 24 and 36 hours. Click here for file

Additional file 4: Figure S4 CXCL12 enhances CXCR4- independent cell signaling. 4T1 breast cancer cells were serum starved for 4 hours and stimulated with TC-14012 (CXCR4 agonist, 30 μ*M*) for different time periods, as indicated, at 37°C. After treatment, cells were washed, lysed, and analyzed for Phospho STAT3, STAT3, Phospho-ERK (p-ERK), and ERK with immunoblotting. Click here for file

Additional file 5: Figure S5 CXCL12 enhances STAT3-mediated cell migration. 4T1 cells were pretreated for 4 hours with vehicle or S31-201 (10 μ*M*), plated on the top chamber of 8-μm-pore polycarbonate membrane filters, and medium in the absence or presence of CXCL12 (100 ng/ml) was placed in the lower chamber. After 12 hours of incubation, cells that migrated across the filter toward medium with or without CXCL12 (100 ng/ml) were fixed, stained, and counted by using bright-field microscopy in five random fields. *P < 0.05 versus none, and ^##^*P* < 0.01 versus control. Click here for file

Additional file 6: Figure S6 Reduced STAT3 activation in 4T1.2 tumors treated with CXCR7 or STAT3 inhibitor. **(A)** Tumors from mice used in the experiment presented in Figure 2 were subjected to IHC staining for p-STAT3 (40×). The pSTAT3-stained cells were counted in four different fields by using a bright-field microscope in each experimental group, and the average was calculated. **(B)** Bars represent the mean ± SD of number of pSTAT3 cells to that of total cells. Scale bars, 0.02 mm. ^***^P < 0.001 versus control. Click here for file

## References

[B1] Kan N Kuwata K Mise K Kodama H Effective therapeutic regimens for patients with triple-negative (ER/PgR/HER2-negative) metastatic breast cancer Gan To Kagaku Ryoho 2010 37 1259 1264 20647706

[B2] Guarneri V Conte P Metastatic breast cancer: therapeutic options according to molecular subtypes and prior adjuvant therapy Oncologist 2009 14 645 656 doi:10.1634/theoncologist.2009-0078 10.1634/theoncologist.2009-0078 19608638

[B3] Fernandez Y Cueva J Palomo AG Ramos M de Juan A Calvo L Garcia-Mata J Garcia-Teijido P Pelaez I Garcia-Estevez L Novel therapeutic approaches to the treatment of metastatic breast cancer Cancer Treat Rev 2010 36 33 42 doi:10.1016/j.ctrv.2009.10.001 10.1016/j.ctrv.2009.10.001 19883980

[B4] Nasser MW Qamri Z Deol YS Smith D Shilo K Zou X Ganju RK Crosstalk between chemokine receptor CXCR4 and cannabinoid receptor CB2 in modulating breast cancer growth and invasion PLoS One 2011 6 e23901 doi:10.1371/journal.pone.0023901PONE-D-11-12697 10.1371/journal.pone.0023901 21915267 PMC3168464

[B5] Kang H Mansel RE Jiang WG Genetic manipulation of stromal cell-derived factor-1 attests the pivotal role of the autocrine SDF-1-CXCR4 pathway in the aggressiveness of breast cancer cells Int J Oncol 2005 26 1429 1434 15809737

[B6] Liang Z Yoon Y Votaw J Goodman MM Williams L Shim H Silencing of CXCR4 blocks breast cancer metastasis Cancer Res 2005 65 967 971 15705897 PMC3734941

[B7] Smith MC Luker KE Garbow JR Prior JL Jackson E Piwnica-Worms D Luker GD CXCR4 regulates growth of both primary and metastatic breast cancer Cancer Res 2004 64 8604 8612 doi:10.1158/0008-5472.CAN-04-1844 10.1158/0008-5472.CAN-04-1844 15574767

[B8] Helbig G Christopherson KW 2nd Bhat-Nakshatri P Kumar S Kishimoto H Miller KD Broxmeyer HE Nakshatri H NF-kappaB promotes breast cancer cell migration and metastasis by inducing the expression of the chemokine receptor CXCR4 J Biol Chem 2003 278 21631 21638 doi:10.1074/jbc.M300609200M300609200 10.1074/jbc.M300609200 12690099

[B9] Burns JM Summers BC Wang Y Melikian A Berahovich R Miao Z Penfold ME Sunshine MJ Littman DR Kuo CJ Wei K McMaster BE Wright K Howard MC Schall TJ A novel chemokine receptor for SDF-1 and I-TAC involved in cell survival, cell adhesion, and tumor development J Exp Med 2006 203 2201 2213 doi:10.1084/jem.20052144 10.1084/jem.20052144 16940167 PMC2118398

[B10] Wang J Shiozawa Y Wang Y Jung Y Pienta KJ Mehra R Loberg R Taichman RS The role of CXCR7/RDC1 as a chemokine receptor for CXCL12/SDF-1 in prostate cancer J Biol Chem 2008 283 4283 4294 doi:10.1074/jbc.M707465200 10.1074/jbc.M707465200 18057003

[B11] Hawkins OE Richmond A The dynamic yin-yang interaction of CXCR4 and CXCR7 in breast cancer metastasis Breast Cancer Res 2012 14 103 doi:10.1186/bcr3092 10.1186/bcr3092 22293321 PMC3496126

[B12] Sierro F Biben C Martinez-Munoz L Mellado M Ransohoff RM Li M Woehl B Leung H Groom J Batten M Harvey RP Martinez AC Mackay CR Mackay F Disrupted cardiac development but normal hematopoiesis in mice deficient in the second CXCL12/SDF-1 receptor, CXCR7 Proc Natl Acad Sci U S A 2007 104 14759 14764 doi:10.1073/pnas.0702229104 10.1073/pnas.0702229104 17804806 PMC1976222

[B13] Miao Z Luker KE Summers BC Berahovich R Bhojani MS Rehemtulla A Kleer CG Essner JJ Nasevicius A Luker GD Howard MC Schall TJ CXCR7 (RDC1) promotes breast and lung tumor growth in vivo and is expressed on tumor-associated vasculature Proc Natl Acad Sci U S A 2007 104 15735 15740 doi:10.1073/pnas.0610444104 10.1073/pnas.0610444104 17898181 PMC1994579

[B14] Ray P Lewin SA Mihalko LA Lesher-Perez SC Takayama S Luker KE Luker GD Secreted CXCL12 (SDF-1) forms dimers under physiological conditions Biochem J 2012 442 433 442 doi:10.1042/BJ20111341 10.1042/BJ20111341 22142194 PMC4419379

[B15] Decaillot FM Kazmi MA Lin Y Ray-Saha S Sakmar TP Sachdev P CXCR7/CXCR4 heterodimer constitutively recruits beta-arrestin to enhance cell migration J Biol Chem 2011 286 32188 32197 doi:10.1074/jbc.M111.277038 10.1074/jbc.M111.277038 21730065 PMC3173186

[B16] Rajagopal S Kim J Ahn S Craig S Lam CM Gerard NP Gerard C Lefkowitz RJ Beta-arrestin- but not G protein-mediated signaling by the “decoy” receptor CXCR7 Proc Natl Acad Sci U S A 2010 107 628 632 doi:10.1073/pnas.0912852107 10.1073/pnas.0912852107 20018651 PMC2818968

[B17] Zabel BA Lewen S Berahovich RD Jaen JC Schall TJ The novel chemokine receptor CXCR7 regulates trans-endothelial migration of cancer cells Mol Cancer 2011 10 73 doi:10.1186/1476-4598-10-73 10.1186/1476-4598-10-73 21672222 PMC3123309

[B18] Hernandez L Magalhaes MA Coniglio SJ Condeelis JS Segall JE Opposing roles of CXCR4 and CXCR7 in breast cancer metastasis Breast Cancer Res 2011 13 R128 doi:10.1186/bcr3074 10.1186/bcr3074 22152016 PMC3326570

[B19] Hattermann K Held-Feindt J Lucius R Muerkoster SS Penfold ME Schall TJ Mentlein R The chemokine receptor CXCR7 is highly expressed in human glioma cells and mediates antiapoptotic effects Cancer Res 2010 70 3299 3308 doi:10.1158/0008-5472.CAN-09-3642 10.1158/0008-5472.CAN-09-3642 20388803

[B20] Saini MK Vaish V Sanyal SN Role of cytokines and Jak3/Stat3 signaling in the 1,2-dimethylhydrazine dihydrochloride-induced rat model of colon carcinogenesis: early target in the anticancer strategy Eur J Cancer Prev 2013 22 215 228 doi:10.1097/CEJ.0b013e328358493200008469-201305000-00003 10.1097/CEJ.0b013e3283584932 23514809

[B21] Wang L Lee HK Seo IA Shin YK Lee KY Park HT Cell type-specific STAT3 activation by gp130-related cytokines in the peripheral nerves Neuroreport 2009 20 663 668 doi:10.1097/WNR.0b013e32832a09f8 10.1097/WNR.0b013e32832a09f8 19349921

[B22] Alonzi T Middleton G Wyatt S Buchman V Betz UA Muller W Musiani P Poli V Davies AM Role of STAT3 and PI 3-kinase/Akt in mediating the survival actions of cytokines on sensory neurons Mol Cell Neurosci 2001 18 270 282 doi:10.1006/mcne.2001.1018S1044743101910188 10.1006/mcne.2001.1018 11591128

[B23] Niu G Wright KL Huang M Song L Haura E Turkson J Zhang S Wang T Sinibaldi D Coppola D Heller R Ellis LM Karras J Bromberg J Pardoll D Jove R Yu H Constitutive Stat3 activity up-regulates VEGF expression and tumor angiogenesis Oncogene 2002 21 2000 2008 doi:10.1038/sj.onc.1205260 10.1038/sj.onc.1205260 11960372

[B24] Ranger JJ Levy DE Shahalizadeh S Hallett M Muller WJ Identification of a Stat3-dependent transcription regulatory network involved in metastatic progression Cancer Res 2009 69 6823 6830 doi:10.1158/0008-5472.CAN-09-1684 10.1158/0008-5472.CAN-09-1684 19690134 PMC2841985

[B25] Hsieh FC Cheng G Lin J Evaluation of potential Stat3-regulated genes in human breast cancer Biochem Biophys Res Commun 2005 335 292 299 doi:10.1016/j.bbrc.2005.07.075 10.1016/j.bbrc.2005.07.075 16081048

[B26] Clarkson RW Boland MP Kritikou EA Lee JM Freeman TC Tiffen PG Watson CJ The genes induced by signal transducer and activators of transcription (STAT)3 and STAT5 in mammary epithelial cells define the roles of these STATs in mammary development Mol Endocrinol 2006 20 675 685 doi:10.1210/me.2005-0392 10.1210/me.2005-0392 16293640

[B27] Allavena P Sica A Solinas G Porta C Mantovani A The inflammatory micro-environment in tumor progression: the role of tumor-associated macrophages Crit Rev Oncol Hematol 2008 66 1 9 doi:10.1016/j.critrevonc.2007.07.004 10.1016/j.critrevonc.2007.07.004 17913510

[B28] Pollard JW Tumour-educated macrophages promote tumour progression and metastasis Nat Rev Cancer 2004 4 71 78 doi:10.1038/nrc1256nrc1256 10.1038/nrc1256 14708027

[B29] Lin EY Nguyen AV Russell RG Pollard JW Colony-stimulating factor 1 promotes progression of mammary tumors to malignancy J Exp Med 2001 193 727 740 10.1084/jem.193.6.727 11257139 PMC2193412

[B30] Lin EY Pollard JW Tumor-associated macrophages press the angiogenic switch in breast cancer Cancer Res 2007 67 5064 5066 doi:10.1158/0008-5472.CAN-07-0912 10.1158/0008-5472.CAN-07-0912 17545580

[B31] Zeisberger SM Odermatt B Marty C Zehnder-Fjallman AH Ballmer-Hofer K Schwendener RA Clodronate-liposome-mediated depletion of tumour-associated macrophages: a new and highly effective antiangiogenic therapy approach Br J Cancer 2006 95 272 281 doi:10.1038/sj.bjc.6603240 10.1038/sj.bjc.6603240 16832418 PMC2360657

[B32] Sica A Allavena P Mantovani A Cancer related inflammation: the macrophage connection Cancer Lett 2008 267 204 215 doi:10.1016/j.canlet.2008.03.028 10.1016/j.canlet.2008.03.028 18448242

[B33] Eckhardt BL Parker BS van Laar RK Restall CM Natoli AL Tavaria MD Stanley KL Sloan EK Moseley JM Anderson RL Genomic analysis of a spontaneous model of breast cancer metastasis to bone reveals a role for the extracellular matrix Mol Cancer Res 2005 3 1 13 15671244

[B34] Lelekakis M Moseley JM Martin TJ Hards D Williams E Ho P Lowen D Javni J Miller FR Slavin J Anderson RL A novel orthotopic model of breast cancer metastasis to bone Clin Exp Metastasis 1999 17 163 170 10.1023/A:1006689719505 10411109

[B35] Balasubramanian A Ganju RK Groopman JE Hepatitis C virus and HIV envelope proteins collaboratively mediate interleukin-8 secretion through activation of p38 MAP kinase and SHP2 in hepatocytes J Biol Chem 2003 278 35755 35766 doi:10.1074/jbc.M302889200M302889200 10.1074/jbc.M302889200 12824191

[B36] Fernandis AZ Cherla RP Ganju RK Differential regulation of CXCR4-mediated T-cell chemotaxis and mitogen-activated protein kinase activation by the membrane tyrosine phosphatase, CD45 J Biol Chem 2003 278 9536 9543 doi:10.1074/jbc.M211803200M211803200 10.1074/jbc.M211803200 12519755

[B37] Munshi N Balasubramanian A Koziel M Ganju RK Groopman JE Hepatitis C and human immunodeficiency virus envelope proteins cooperatively induce hepatocytic apoptosis via an innocent bystander mechanism J Infect Dis 2003 188 1192 1204 doi:10.1086/378643 10.1086/378643 14551890

[B38] Qamri Z Preet A Nasser MW Bass CE Leone G Barsky SH Ganju RK Synthetic cannabinoid receptor agonists inhibit tumor growth and metastasis of breast cancer Mol Cancer Ther 2009 8 3117 3129 doi:10.1158/1535-7163.MCT-09-0448 10.1158/1535-7163.MCT-09-0448 19887554 PMC4128286

[B39] Fernandis AZ Prasad A Band H Klosel R Ganju RK Regulation of CXCR4-mediated chemotaxis and chemoinvasion of breast cancer cells Oncogene 2004 23 157 167 doi:10.1038/sj.onc.12069101206910 10.1038/sj.onc.1206910 14712221

[B40] Nasser MW Qamri Z Deol YS Ravi J Powell CA Trikha P Schwendener RA Bai XF Shilo K Zou X Leone G Wolf R Yuspa SH Ganju RK S100A7 enhances mammary tumorigenesis through upregulation of inflammatory pathways Cancer Res 2012 72 604 615 doi:10.1158/0008-5472.CAN-11-0669 10.1158/0008-5472.CAN-11-0669 22158945 PMC3271140

[B41] Sneh A Deol YS Ganju A Shilo K Rosol TJ Nasser MW Ganju RK Differential role of psoriasin (S100A7) in estrogen receptor alpha positive and negative breast cancer cells occur through actin remodeling Breast Cancer Res Treat 2013 136 727 739 doi:10.1007/s10549-013-2491-4 23535840 10.1007/s10549-013-2491-4 PMC4070432

[B42] Minn AJ Gupta GP Siegel PM Bos PD Shu W Giri DD Viale A Olshen AB Gerald WL Massague J Genes that mediate breast cancer metastasis to lung Nature 2005 436 518 524 doi:10.1038/nature03799 10.1038/nature03799 16049480 PMC1283098

[B43] Thrane S Lykkesfeldt AE Larsen MS Sorensen BS Yde CW Estrogen receptor alpha is the major driving factor for growth in tamoxifen-resistant breast cancer and supported by HER/ERK signaling Breast Cancer Res Treat 2013 139 71 80 doi:10.1007/s10549-013-2485-2 10.1007/s10549-013-2485-2 23609470

[B44] Tarkkonen K Ruohola J Harkonen P Fibroblast growth factor 8 induced downregulation of thrombospondin 1 is mediated by the MEK/ERK and PI3K pathways in breast cancer cells Growth Factors 2010 28 256 267 doi:10.3109/08977191003745480 10.3109/08977191003745480 20370578

[B45] Frogne T Benjaminsen RV Sonne-Hansen K Sorensen BS Nexo E Laenkholm AV Rasmussen LM Riese DJ 2nd de Cremoux P Stenvang J Lykkesfeldt AE Activation of ErbB3, EGFR and Erk is essential for growth of human breast cancer cell lines with acquired resistance to fulvestrant Breast Cancer Res Treat 2009 114 263 275 doi:10.1007/s10549-008-0011-8 10.1007/s10549-008-0011-8 18409071 PMC2764248

[B46] Song L Turkson J Karras JG Jove R Haura EB Activation of Stat3 by receptor tyrosine kinases and cytokines regulates survival in human non-small cell carcinoma cells Oncogene 2003 22 4150 4165 doi:10.1038/sj.onc.12064791206479 10.1038/sj.onc.1206479 12833138

[B47] Zhang SS Liu MG Kano A Zhang C Fu XY Barnstable CJ STAT3 activation in response to growth factors or cytokines participates in retina precursor proliferation Exp Eye Res 2005 81 103 115 doi:10.1016/j.exer.2005.01.016 10.1016/j.exer.2005.01.016 15978261

[B48] Stein M Keshav S Harris N Gordon S Interleukin 4 potently enhances murine macrophage mannose receptor activity: a marker of alternative immunologic macrophage activation J Exp Med 1992 176 287 292 10.1084/jem.176.1.287 1613462 PMC2119288

[B49] Utsunomiya Y Omura K Yokoo T Imasawa T Kawamura T Abe A Hirano K Mitarai T Maruyama N Sakai O Macrophage-colony stimulating factor (M-CSF) enhances proteinuria and recruitment of macrophages into the glomerulus in experimental murine nephritis Clin Exp Immunol 1996 106 286 296 10.1046/j.1365-2249.1996.d01-831.x 8918575 PMC2200600

[B50] Le Meur Y Tesch GH Hill PA Mu W Foti R Nikolic-Paterson DJ Atkins RC Macrophage accumulation at a site of renal inflammation is dependent on the M-CSF/c-fms pathway J Leukoc Biol 2002 72 530 537 12223521

[B51] Dechow TN Pedranzini L Leitch A Leslie K Gerald WL Linkov I Bromberg JF Requirement of matrix metalloproteinase-9 for the transformation of human mammary epithelial cells by Stat3-C Proc Natl Acad Sci U S A 2004 101 10602 10607 doi:10.1073/pnas.04041001010404100101 10.1073/pnas.0404100101 15249664 PMC489981

[B52] Wiseman BS Werb Z Stromal effects on mammary gland development and breast cancer Science 2002 296 1046 1049 doi:10.1126/science.1067431296/5570/1046 10.1126/science.1067431 12004111 PMC2788989

[B53] Chen Q Zhang XH Massague J Macrophage binding to receptor VCAM-1 transmits survival signals in breast cancer cells that invade the lungs Cancer Cell 2011 20 538 549 doi:10.1016/j.ccr.2011.08.025 10.1016/j.ccr.2011.08.025 22014578 PMC3293160

[B54] The Cancer Genome Atlas Network Comprehensive molecular portraits of human breast tumours Nature 2012 490 61 70 doi:10.1038/nature11412 10.1038/nature11412 23000897 PMC3465532

[B55] Cerami E Gao J Dogrusoz U Gross BE Sumer SO Aksoy BA Jacobsen A Byrne CJ Heuer ML Larsson E Antipin Y Reva B Goldberg AP Sander C Schultz N The cBio cancer genomics portal: an open platform for exploring multidimensional cancer genomics data Cancer Discov 2012 2 401 404 doi:10.1158/2159-8290.CD-12-0095 10.1158/2159-8290.CD-12-0095 22588877 PMC3956037

[B56] Balkwill F Cancer and the chemokine network Nat Rev Cancer 2004 4 540 550 doi:10.1038/nrc1388nrc1388 10.1038/nrc1388 15229479

[B57] Kang H Watkins G Douglas-Jones A Mansel RE Jiang WG The elevated level of CXCR4 is correlated with nodal metastasis of human breast cancer Breast 2005 14 360 367 doi:10.1016/j.breast.2004.12.007 10.1016/j.breast.2004.12.007 16216737

[B58] Teicher BA Fricker SP CXCL12 (SDF-1)/CXCR4 pathway in cancer Clin Cancer Res 2010 16 2927 2931 doi:10.1158/1078-0432.CCR-09-2329 10.1158/1078-0432.CCR-09-2329 20484021

[B59] Koshiba T Hosotani R Miyamoto Y Ida J Tsuji S Nakajima S Kawaguchi M Kobayashi H Doi R Hori T Fujii N Imamura M Expression of stromal cell-derived factor 1 and CXCR4 ligand receptor system in pancreatic cancer: a possible role for tumor progression Clin Cancer Res 2000 6 3530 3535 10999740

[B60] Lapteva N Yang AG Sanders DE Strube RW Chen SY CXCR4 knockdown by small interfering RNA abrogates breast tumor growth in vivo Cancer Gene Ther 2005 12 84 89 doi:10.1038/sj.cgt.7700770 10.1038/sj.cgt.7700770 15472715

[B61] Darash-Yahana M Pikarsky E Abramovitch R Zeira E Pal B Karplus R Beider K Avniel S Kasem S Galun E Peled A Role of high expression levels of CXCR4 in tumor growth, vascularization, and metastasis FASEB J 2004 18 1240 1242 doi:10.1096/fj.03-0935fje03-0935fje 15180966 10.1096/fj.03-0935fje

[B62] Luker KE Gupta M Steele JM Foerster BR Luker GD Imaging ligand-dependent activation of CXCR7 Neoplasia 2009 11 1022 1035 19794961 10.1593/neo.09724 PMC2745668

[B63] Luker KE Steele JM Mihalko LA Ray P Luker GD Constitutive and chemokine-dependent internalization and recycling of CXCR7 in breast cancer cells to degrade chemokine ligands Oncogene 2010 29 4599 4610 doi:10.1038/onc.2010.212 10.1038/onc.2010.212 20531309 PMC3164491

[B64] Xue TC Chen RX Ye SL Sun RX Chen J Tang ZY Different expressions of chemokine receptors in human hepatocellular carcinoma cell lines with different metastatic potentials Zhonghua Gan Zang Bing Za Zhi 2007 15 261 265 17456312

[B65] Xue TC Chen RX Ren ZG Zou JH Tang ZY Ye SL Transmembrane receptor CXCR7 increases the risk of extrahepatic metastasis of relatively well-differentiated hepatocellular carcinoma through upregulation of osteopontin Oncol Rep 2013 30 105 110 doi:10.3892/or.2013.2442 23636305 10.3892/or.2013.2442

[B66] Takanami I Ohnishi H Study of surgical resection of sternal metastasis from carcinoma of the breast Gan No Rinsho 1989 35 1735 1738 2607606

[B67] Zhang YW Zhang ZX Miao ZH Ding J The telomeric protein TRF2 is critical for the protection of A549 cells from both telomere erosion and DNA double-strand breaks driven by salvicine Mol Pharmacol 2008 73 824 832 doi:10.1124/mol.107.039081 18025071 10.1124/mol.107.039081

[B68] Clevenger CV Roles and regulation of stat family transcription factors in human breast cancer Am J Pathol 2004 165 1449 1460 doi:10.1016/S0002-9440(10)63403-7 10.1016/S0002-9440(10)63403-7 15509516 PMC1618660

[B69] Li N Grivennikov SI Karin M The unholy trinity: inflammation, cytokines, and STAT3 shape the cancer microenvironment Cancer Cell 2011 19 429 431 doi:10.1016/j.ccr.2011.03.018 10.1016/j.ccr.2011.03.018 21481782 PMC3111086

[B70] O'Shea JJ Pesu M Borie DC Changelian PS A new modality for immunosuppression: targeting the JAK/STAT pathway Nat Rev Drug Discov 2004 3 555 564 doi:10.1038/nrd1441nrd1441 10.1038/nrd1441 15232577

[B71] Chang Q Bournazou E Sansone P Berishaj M Gao SP Daly L Wels J Theilen T Granitto S Zhang X Cotari J Alpaugh ML de Stanchina E Manova K Li M Bonafe M Ceccarelli C Taffurelli M Santini D Altan-Bonnet G Kaplan R Norton L Nishimoto N Huszar D Lyden D Bromberg J The IL-6/JAK/Stat3 feed-forward loop drives tumorigenesis and metastasis Neoplasia 2013 15 848 862 23814496 10.1593/neo.13706 PMC3689247

[B72] Weinstat-Saslow D Merino MJ Manrow RE Lawrence JA Bluth RF Wittenbel KD Simpson JF Page DL Steeg PS Overexpression of cyclin D mRNA distinguishes invasive and in situ breast carcinomas from non-malignant lesions Nat Med 1995 1 1257 1260 10.1038/nm1295-1257 7489405

[B73] Kruse J von Bernstorff W Evert K Albers N Hadlich S Hagemann S Gunther C van Rooijen N Heidecke CD Partecke LI Macrophages promote tumour growth and liver metastasis in an orthotopic syngeneic mouse model of colon cancer Int J Colorectal Dis 2013 28 1337 1349 doi:10.1007/s00384-013-1703-z 10.1007/s00384-013-1703-z 23657400

[B74] Woodhouse EC Chuaqui RF Liotta LA General mechanisms of metastasis Cancer 1997 80 1529 1537 doi:10.1002/(SICI)1097-0142(19971015)80:8+<1529::AID-CNCR2>3.0.CO;2-F 10.1002/(SICI)1097-0142(19971015)80:8+<1529::AID-CNCR2>3.0.CO;2-F 9362419

[B75] van der Bij GJ Bogels M Oosterling SJ Kroon J Schuckmann DT de Vries HE Meijer S Beelen RH van Egmond M Tumor infiltrating macrophages reduce development of peritoneal colorectal carcinoma metastases Cancer Lett 2008 262 77 86 doi:10.1016/j.canlet.2007.11.040 10.1016/j.canlet.2007.11.040 18187256

