# Supplementary figures and images for: C-X-C motif chemokine 12/C-X-C chemokine receptor type 7 signaling regulates breast cancer growth and metastasis by modulating the tumor microenvironment
Source: Breast Cancer Res. 2014 May 29;16(3):R54. doi: 10.1186/bcr3665 (PMC4076630; doi:10.1186/bcr3665)

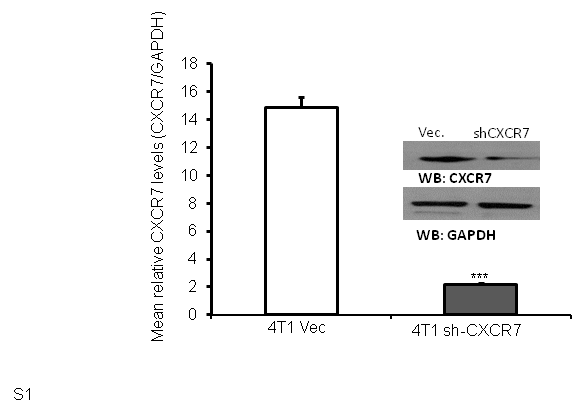

Supplement: Additional file 1: Figure S1 — CXCR7 expression in 4T1 Vec and sh-RNA downregulated cells. 4T1 Vec and 4T1 sh-CXCR7 cell lines were lysed and analyzed with Western blotting for CXCR7 and GAPDH expression. Data represent the mean ± SD per experimental group. ***P < 0.001 versus vector control. [file bcr3665-S1.tiff]

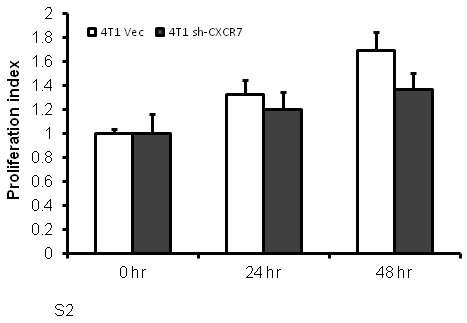

Supplement: Additional file 2: Figure S2 — CXCR7 downregulation did not inhibit proliferation in vitro. 4T1 Vec and 4T1 sh-CXCR7 cell lines were seeded at a density of 5,000 cells per well in 96-well plates and allowed to grow for 24 to 48 hours in SFM. Cell viability was measured by using the MTT assay (Roche), based on the absorbance reading at 570 nm with respect to the control. [file bcr3665-S2.tiff]

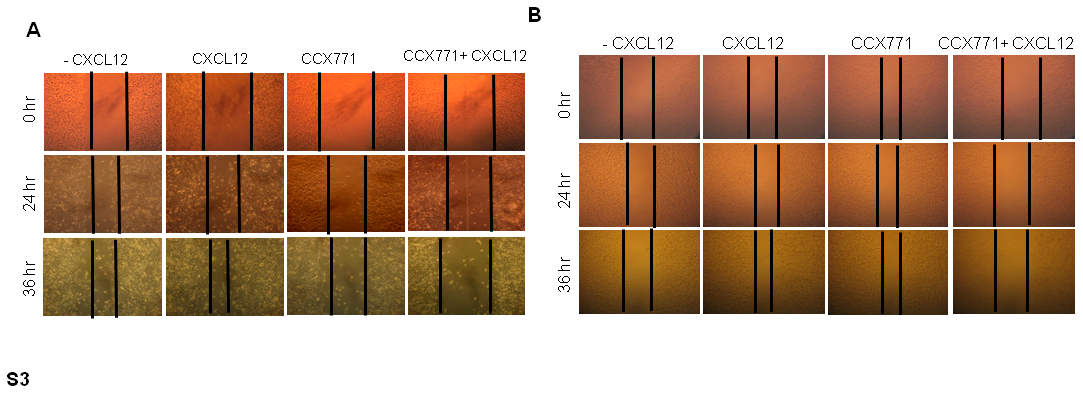

Supplement: Additional file 3: Figure S3 — CXCL12 enhances CXCR7- mediated cell migration. 4T1 (A) and 4T1.2 (B) cells treated with CCX771 (1 μM) were grown for confluence in incomplete medium in six-well plates, and then a scratch was made with a 200-μl pipette tip to make wounds; the closure of the wounds was monitored in the presence or absence of CXCL12 (100 ng/ml) by microscopy after 24 and 36 hours. [file bcr3665-S3.tiff]

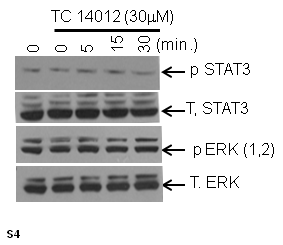

Supplement: Additional file 4: Figure S4 — CXCL12 enhances CXCR4- independent cell signaling. 4T1 breast cancer cells were serum starved for 4 hours and stimulated with TC-14012 (CXCR4 agonist, 30 μM) for different time periods, as indicated, at 37°C. After treatment, cells were washed, lysed, and analyzed for Phospho STAT3, STAT3, Phospho-ERK (p-ERK), and ERK with immunoblotting. [file bcr3665-S4.tiff]

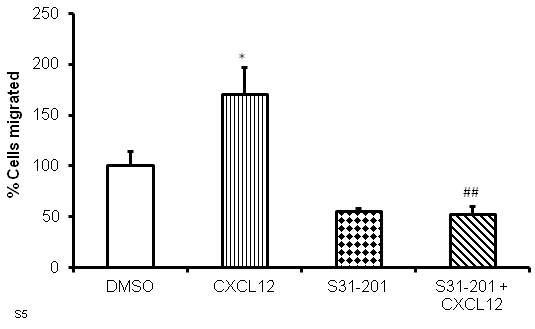

Supplement: Additional file 5: Figure S5 — CXCL12 enhances STAT3-mediated cell migration. 4T1 cells were pretreated for 4 hours with vehicle or S31-201 (10 μM), plated on the top chamber of 8-μm-pore polycarbonate membrane filters, and medium in the absence or presence of CXCL12 (100 ng/ml) was placed in the lower chamber. After 12 hours of incubation, cells that migrated across the filter toward medium with or without CXCL12 (100 ng/ml) were fixed, stained, and counted by using bright-field microscopy in five random fields. *P < 0.05 versus none, and ##P < 0.01 versus control. [file bcr3665-S5.tiff]

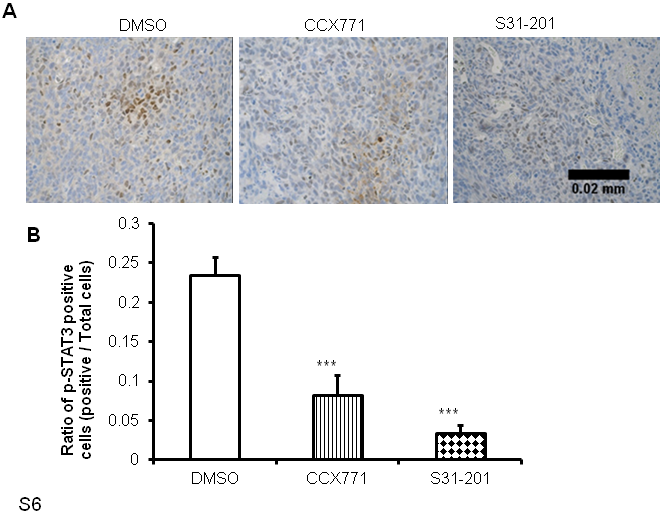

Supplement: Additional file 6: Figure S6 — Reduced STAT3 activation in 4T1.2 tumors treated with CXCR7 or STAT3 inhibitor. (A) Tumors from mice used in the experiment presented in Figure 2 were subjected to IHC staining for p-STAT3 (40×). The pSTAT3-stained cells were counted in four different fields by using a bright-field microscope in each experimental group, and the average was calculated. (B) Bars represent the mean ± SD of number of pSTAT3 cells to that of total cells. Scale bars, 0.02 mm. ***P < 0.001 versus control. [file bcr3665-S6.tiff]
